# Characterization of SN38-resistant T47D breast cancer cell sublines overexpressing BCRP, MRP1, MRP2, MRP3, and MRP4

**DOI:** 10.1186/s12885-022-09446-y

**Published:** 2022-04-23

**Authors:** Hee-Jeong Lee, Cheol-Hee Choi

**Affiliations:** 1 Department of Hemato-Oncology, Chosun University Hospital, Gwangju, 501-759 Korea; 2 Research Center for Resistant Cells, Chosun University, Gwangju, 501-759 Korea; 3 Department of Pharmacology, Chosun University Medical School, Gwangju, 501-759 Korea

**Keywords:** Breast cancer, T47D, SN-38, Multidrug resistance, Breast cancer resistance protein, Epigenetic silencing, Chemosensitizer

## Abstract

**Background:**

Although several novel resistant breast cancer cell lines have been established, only a few resistant breast cancer cell lines overexpress breast cancer resistance proteins (*BCRP*). The aim of this study was to establish new resistant breast cancer cell lines overexpressing *BCRP* using SN38 (7-ethyl-10-hydroxycamptothecin), an active metabolite of irinotecan and was to discover genes and mechanisms associated with multidrug resistance.

**Methods:**

SN38-resistant T47D breast cancer cell sublines were selected from the wild-type T47D cells by gradually increasing SN38 concentration. The sensitivity of the cells to anti-cancer drugs was assessed by 3-(4,5-methylthiazol-2-yl)-2,5-diphenyl-tetrazolium bromide (MTT) assay. Expression profiles of the resistance-related transporters were examined using RT-qPCR, and western blot analysis. Intracellular fluorescent dye accumulation in the resistant cells was determined using flow cytometry.

**Results:**

The SN38-resistant T47D breast cancer cell sublines T47D/SN120 and T47D/SN150 were established after long-term exposure (more than 16 months) of wild-type T47D cells to 120 nM and 150 nM SN38, respectively. T47D/SN120 and T47D/SN150 cells were more resistant to SN38 (14.5 and 59.1 times, respectively), irinotecan (1.5 and 3.7 times, respectively), and topotecan (4.9 and 12 times, respectively), than the wild-type parental cells. Both T47D/SN120 and T47D/SN150 sublines were cross-resistant to various anti-cancer drugs. These resistant sublines overexpressed mRNAs of *MRP1, MRP2, MRP3, MRP4,* and *BCRP*. The DNA methylase inhibitor 5-aza-2′-deoxycytidine and the histone deacetylase inhibitor trichostatin A increased the expression levels of *BCRP, MRP1, MRP2, MRP3*, and *MRP4* transcripts in T47D/WT cells. Fluorescent dye accumulation was found to be lower in T47D/SN120 and T47D/SN150 cells, compared to that in T47D/WT cells. However, treatment with known chemosensitizers increased the intracellular fluorescent dye accumulation and sensitivity of anti-tumor agents.

**Conclusion:**

T47D/SN120 and T47D/SN150 cells overexpressed *MRP1, MRP2, MRP3, MRP4*, and *BCRP*, which might be due to the suppression of epigenetic gene silencing via DNA hypermethylation and histone deacetylation. Although these resistant cells present a higher resistance to various anti-cancer drugs than their parental wild-type cells, multidrug resistance was overcome by treatment with chemosensitizers. These SN38 resistant T47D breast cancer cell sublines expressing resistance proteins can be useful for the development of new chemosensitizers.

**Supplementary Information:**

The online version contains supplementary material available at 10.1186/s12885-022-09446-y.

## Background

Breast cancer is not only a frequently diagnosed cancer worldwide, it is also a leading cause of cancer-related death in women [1]. The treatment of breast cancer is largely classified into two categories: local treatment including surgery, radiation, or both modalities; and systemic treatment including cytotoxic chemotherapy, endocrine therapy, biological therapy, or a combination of these. Systemic treatment, which is used in adjuvant, neoadjuvant, and palliative settings, plays an important role in the treatment of breast cancer at various stages. Indeed, systemic agents are effective at the beginning of therapy in 69–95% of primary breast cancers [2–6] and 50% of metastatic cancers [7–9]. However, cancer progression often occurs after a variable duration of chemotherapy, owing to the development of chemotherapy resistance, which is divided into primary resistance and acquired resistance. Primary resistance includes cases that are unresponsive despite the use of appropriate initial chemotherapy, with continued tumor growth during the treatment [10]. Acquired resistance involves cases where the tumor cells initially seem to respond well to the chemotherapy but acquire resistance to the anti-cancer drugs due to repeated exposure [10]; in this case, cells are resistant to some agents of the same class but sensitive to drugs of different classes. However, eventual cross-resistance to multiple anti-cancer drugs of apparently different structures and functions is observed; this phenomenon is known as multidrug resistance (MDR) [11].

The mechanisms and pathways of MDR are complicated and multi-factorial. One of the mechanisms of MDR is associated with the alteration of anti-cancer drug transporter. One of the classic MDR mechanisms involves decreased drug accumulation due to increased expression of drug efflux pump in the tumor cell membrane [12]. This MDR phenotype is mediated by ATP (adenosine triphosphate)-binding cassette (ABC) transporters [12], including *P-glycoprotein* (*Pgp*) [13–15] and members of the multidrug resistance protein (*MRP*) family [16–18]. Another novel transporter, breast cancer resistance protein (*BCRP*), has been identified as an ABC half-transporter and is distributed in the placenta and various cancer types [19–22]. Meanwhile, it was reported that a redistribution of the anti-cancer drug from the nucleus to the cytoplasm is related to non-ABC transporters-mediated MDR [23]. Lung resistance protein (*LRP*) is involved in the nucleo-cytoplasmic transport and cytoplasmic sequestration of anti-cancer drugs [24–26]. Another mechanism of MDR is associated with alterations in topoisomerase [27–31].

Based on comprehensive knowledge about the mechanisms of drug resistance, recent studies on the mechanism of resistance to anticancer drugs in breast cancer have been reported [32]. Moreover, exploring the mechanism of MDR and finding molecular targets for drug resistance is important for the development of new therapeutic agents to treat cancer. Thus, further studies are vital for a better understanding of MDR mechanisms in breast cancer and development of various types of resistant cancer cell lines.

Recently, several novel resistant breast cancer cell lines have been established. Notably, although *BCRP* expression is observed in primary breast cancer as well as in normal breast tissue [33–35], only a few resistant breast cancer cell lines overexpress *BCRP* [36–39]. Tumor cell lines resistant to the camptothecin-derived topoisomerase I inhibitor topotecan have been shown to overexpress *BCRP* and display a significant cross-resistance to CPT11, SN38 (7-ethyl-10-hydroxycamptothecin), and 9-aminocamptothecin [28, 40–42]. SN38, an active metabolite of irinotecan, possesses a much stronger cytotoxicity against tumor cells than irinotecan [43, 44] through the inhibition of DNA topoisomerase I [45]. Among breast cancer cell lines, MDA-MB 231 is estrogen receptor (ER)-negative, while T47D and MCF-7 are ER-positive [46]. Recently, SN-38-resistant MCF-7 and MDA-MB-231 sublines have been generated [47]. However, no SN38-resistant breast cancer cell T47D sublines have been developed so far. Therefore, the specific aim of this study was to establish SN38-resistant breast cancer cell sublines, anticipating the overexpression of transporters, such as *BCRP* and to investigate MDR mechanisms. Herein, we describe two SN38-resistant breast cancer cell lines, T47D/SN120 and T47D/SN150, characterized by *MRP1, MRP2, MRP3, MRP4*, and *BCRP* overexpression. These resistant cancer cell sublines will be used to develop chemosensitizers that can reverse the resistance.

## Methods

### Cell culture

The human breast cancer cell line T47D was purchased from the Cancer Research Center in Seoul National University (Seoul, South Korea). The cells were cultured in RPMI-1640 (Gibco BRL Grand Island, NY, USA) supplemented with 10% fetal bovine serum (Sigma-Aldrich, St. Louis, MO, USA). The cells were allowed to adhere to the culture dish and form a monolayer. The cells were sub-cultured once they reached confluence.

### Establishment of the SN38-resistant breast cancer cell sublines

The SN38-resistant breast cancer cell sublines T47D/SN120 and T47D/SN150 were established by gradually increasing the concentration of SN38 from 15 nM (IC_50_ value) to final concentrations of 120 nM (IC_50_ × 8) and 150 nM (IC_50_ × 10), respectively in the parental T47D cells. Stable T47D/SN120 and T47D/SN150 cell lines were obtained in a timeframe of 16 months.

### Calculation of cellular population doubling time

The cells were seeded into 24-well culture plates at a density of 5 × 10^4^ cells per well and incubated at 37 °C for 24 h. The cell numbers were counted every 24 h for 4 days. The cell doubling time (Td) was calculated using the formula: Td = T x log2/(log N_t_- log N_0_), where N_0_ and N_t_ represent the number of cells at the beginning and end of the culture during time T, respectively.

### Chemosensitivity test using MTT assay

The 3-(4,5-methylthiazol-2-yl)-2,5-diphenyl-tetrazolium bromide (MTT; Sigma-Aldrich) assay was performed to assess the sensitivity of T47D/SN120 and T47D/SN150 cells to anti-cancer drugs. 50% inhibitory concentration (IC_50_) was defined as the drug concentration that causes a 50% reduction in the number of cells compared to the untreated control. The IC_50_ values were determined directly from the dose-response curves. Resistance factor (RF) was calculated from the ratios of the IC_50_ values of T47D/SN120 and T47D/SN150 to T47D/WT cells.

### RNA extraction and reverse transcription quantitative polymerase chain reaction (RT-qPCR) assay

Total RNA was extracted from the cells using the RNeasy mini kit (Qiagen, Hilden, Germany). mRNA expression was determined by RT-qPCR and normalized to β-actin mRNA expression level. Genes as well as the conventional and real-time primer pairs are listed in Table 1.

**Table 1:** Primer sequences of PCR

Gene	Primer	Conventional	Real-time (5′- 3′)
β-actin	Sense		GACTATGACTTAGTTGCGTTA
	Antisense		GTTGAACTCTTACATATTCCG
***ABCG2*** (BCRP)	Sense	GCCTACAACTGGCTTAGACT	CAGGTCTGTTGGTCAATCTCAC
	Antisense	GATGATTGTTCGTCCCTGCT	CAGTGTGATGGCAAGGGAAC
***ABCC1*** (MRP1)	Sense	GGTCAGCCCAACTCTCTTG	CTAACCTGGACCTGGAACTG
	Antisense	ACTGAACTCCCTTCCTCCTC	TCAATCAACACTGTAAGCAACC
***ABCC2*** (MRP2)	Sense	TCAGGTTTGCCAGTTATCCG	AACCTCATTCAGACGACCATCC
	Antisense	TGGTTGGTGTCAATCCTCAC	GACCATTACCTTGTCACTGTCC
***ABCC3*** (MRP3)	Sense	CCTGCTACTTGCTCTACCTG	CTCCAAGACAGAGACAGAGGC
	Antisense	ACACCCAGGACCATCTTGA	TGGCCCACGCTGAGATTCTC
***ABCC4*** (MRP4)	Sense	GGGAGAGAACCAGCACTTC	AACCTCTAACCGACATTCCTG
	Antisense	TGCTGTTTCCAAGGCATCT	TCAACATATTACAGCCACCATC

RNA was reverse transcribed using Moloney murine leukemia virus reverse transcriptase (Bethesda Research Laboratories, USA) and an oligo (dT) primer for 1 h at 37 °C. The synthesized cDNA was diluted 1:5 with water and amplified using 2.5 units of Taq DNA polymerase (TaKaRa, Tokyo, Japan) and 10 pmol of each primer, under defined PCR conditions, using a GeneAmp PCR system 9600 (Perkin-Elmer-Cetus, Waltham, MA, USA). After the final cycle, all the PCR products were subjected to a final extension at 72 °C for 5 min. The PCR products were electrophoresed on agarose gel. qPCR was conducted by LightCycler® 2.0 (Roche, USA) using TB Green® Premix Ex Taq (Tli RNaseH Plus) (TaKaRa, Tokyo, Japan). The endpoint used in PCR quantification (Ct) was defined as the PCR cycle number that crosses an arbitrarily placed signal threshold.

### Western blot analysis

Cells were washed with phosphate-buffered saline (PBS) and lysed in 50 mM Tris-HCl (pH 7.4), 250 mM NaCl, 0.5% Triton X**-**100, 10% glycerol, 1 mM DTT, 1 mM phenylmethylsulfonyl fluoride, and protease inhibitor cocktail (Pierce Biotechnology, Rockford, IL, USA). The cell lysates were centrifuged and then resolved by sodium dodecyl sulfate polyacrylamide gel electrophoresis. Western blotting was performed using a slight modification of the method described previously [48]. The membrane was incubated with primary rabbit polyclonal antibodies against *MRP1* (1:1000; Invitrogen, Carlsbad, CA, USA), *MRP2* (1:5000; Sigma-Aldrich), *MRP3* (1:50; Abcam, Cambridge, England), *MRP4* (1:50; Abcam), *BCRP* (1:500; Santa Cruz Biotechnology, Santa Cruz, CA, USA), and GAPDH (1:6000; Santa Cruz Biotechnology, Texas, USA). The membrane was then washed and incubated with horseradish peroxidase-conjugated secondary antibodies (1:2500 for *BCRP* and GAPDH) for 1 h. The signal was then detected using the ECL detection kit (Amersham, Piscataway, NJ, USA). Densities of blots were determined by densitometric analysis using a Kodak Image Station 4000MM (Eastman Kodak, Rochester, NY, USA).

### Fluorescent dye accumulation assay

Cell suspensions (5 × 10^5^ cells) in PBS were exposed to 1 μM rhodamine 123, 50 nM calcein-AM, and 20 μM mitoxantrone at 37 °C for 1 h. Additionally, cells were incubated in the presence of each fluorescent substrate with 10 nM PSC833, 1 mM probenecid, 5 mM probenecid, 200 μM genistein, and 2 mM cyanide in PBS at 37 °C for 1 h. After incubation, cellular fluorescent dye accumulation was determined using a flow cytometer (FACSCalibur, Becton Dickinson, MA, USA), which detected drug fluorescence. A focused argon laser beam (488 nm) excited the cells in a laminar sheath flow, following which the fluorescence emissions at 530 nm (for rhodamine 123 and calcein-AM) and 670 nm (for mitoxantrone) were detected to generate the histogram.

### Screening of chemosensitizers

IC_50_ values of SN38 were obtained in the presence and absence of chemosensitizers for SN38-resistant T47D sublines and the ratios were defined as chemosensitizing index.

### Determination of involvement of epigenetic gene silencing of transporters in the T47D/WT cells

We tested if hypermethylation and deacetylation are involved in epigenetic gene silencing by treating the cells with the DNA methyltransferase inhibitor (2.5 uM 5-aza-2′-deoxycytidine) for 96 h and the histone deacetylase (HDAC) inhibitor (100 ng /ml trichostatin A) for 48 h, respectively.

### Statistical analysis

All experiments were repeated more than three times. Statistical significance of the data was determined using student’s t-test. *P* values less than 0.05 were considered significant.

## Results

### Establishment and characterization of resistant cell lines

SN38-resistant T47D/SN120 and T47D/SN150 cell sublines were established from the wild-type T47D cells following long-term exposure (of more than 16 months) to 120 nM and 150 nM SN38, respectively. Microscopic observation showed some distinct features in the SN38-resistant T47D sublines compared to their parental cell line. In monolayer, T47D/WT cells were relatively consistent in size and shape, while the resistant cells presented a spindle-shaped morphology and were smaller in size (Supplementary Fig. 1). Interestingly, the doubling times of T47D/SN120 and T47D/SN150 cell lines were shorter as 38.0 ± 1.33 h and 46.7 ± 2.64 h, respectively, than that of the parental cell lines (70.5 ± 3.84 h) (Supplementary Fig. 2).

### Cross-resistance to other anti-cancer drugs of resistant cell lines

MTT assay showed that T47D/SN120 and T47D/SN150 cells were more resistant to SN38 (14.5 and 59.1 times, respectively), irinotecan (1.5 and 3.7 times, respectively), and topotecan (4.9 and 12 times, respectively) as compared to the wild-type drug-sensitive parental cells. In addition to topoisomerase I inhibitors, both T47D/SN120 and T47D/SN150 sublines were cross-resistant to various anti-cancer drugs that are used in breast cancer treatment, including microtubule inhibitors (paclitaxel and vinblastine), anti-metabolites (5-fluorouracil), topoisomerase II inhibitors (doxorubicin and mitoxantrone), estrogen receptor blockers (endoxifen), and a tyrosine kinase inhibitor (gefitinib) (Table 2).

**Table 2:** The sensitivity of the T47D/WT, T47D/SN120 and T47D/SN150 cells to SN38 and other anticancer drugs. Drugs are sorted according to the order of relative resistance of T47D/SN120 cells to T47D/WT cells

Drug	T47D/WT	T47D/SN120	T47D/SN150
	IC_50_ μg/mL value (RF)		
Paclitaxel	0.007 ± 0.0004 (1)	15.54 ± 3.38 (2220)	41.45 ± 1.52 (5921)
Vinblastine	0.017 ± 0.0004 (1)	33.04 ± 1.12 (1943)	39.96 ± 3.10 (2350)
SN38	0.017 ± 0.0037 (1)	0.25 ± 0.01 (14.5)	1.00 ± 0.20 (59.1)
Doxorubicin	0.056 ± 0.0095 (1)	0.59 ± 0.08 (10.4)	1.07 ± 0.08 (19.1)
Topotecan	0.16 ± 0.036 (1)	0.79 ± 0.23 (4.9)	1.92 ± 0.18 (12.0)
Gefitinib	23.53 ± 0.745 (1)	97.11 ± 2.91 (4.1)	97.21 ± 2.27 (4.1)
Mitoxantrone	0.057 ± 0.032 (1)	0.20 ± 0.04 (3.4)	1.51 ± 0.22 (26.4)
Endoxifen	25.83 ± 4.318 (1)	79.34 ± 0.17 (3.1)	68.05 ± 2.09 (2.6)
5-FU	2.89 ± 0.214 (1)	8.19 ± 0.77 (2.8)	9.83 ± 0.39 (3.4)
Irinotecan	10.24 ± 0.399 (1)	14.98 ± 0.36 (1.5)	38.03 ± 2.37 (3.7)
Cisplatin	118.84 ± 27.13 (1)	159.51 ± 7.82 (1.3)	194.17 ± 8.42 (1.6)
Tamoxifen	46.06 ± 3.512 (1)	59.65 ± 2.44 (1.3)	66.64 ± 2.75 (1.4)
Methotrexate	53.91 ± 6.316 (1)	57.72 ± 2.38 (1.1)	43.48 ± 8.46 (0.8)

*RF* Resistance factor: Relative resistance fold as compared with T47D/WT

As shown in Table 2, both resistant sublines were highly resistant to paclitaxel and vinblastine. Additionally, T47D/SN150 cells showed a high resistance to SN38, mitoxantrone and doxorubicin and moderate resistance to topotecan. However, the sensitivity to gefinitib, endoxifen, 5-FU and methotrexate appeared similar between both resistant sublines.

### Expression profiles of transporters in the T47D/SN sublines

**Fig. 1:**
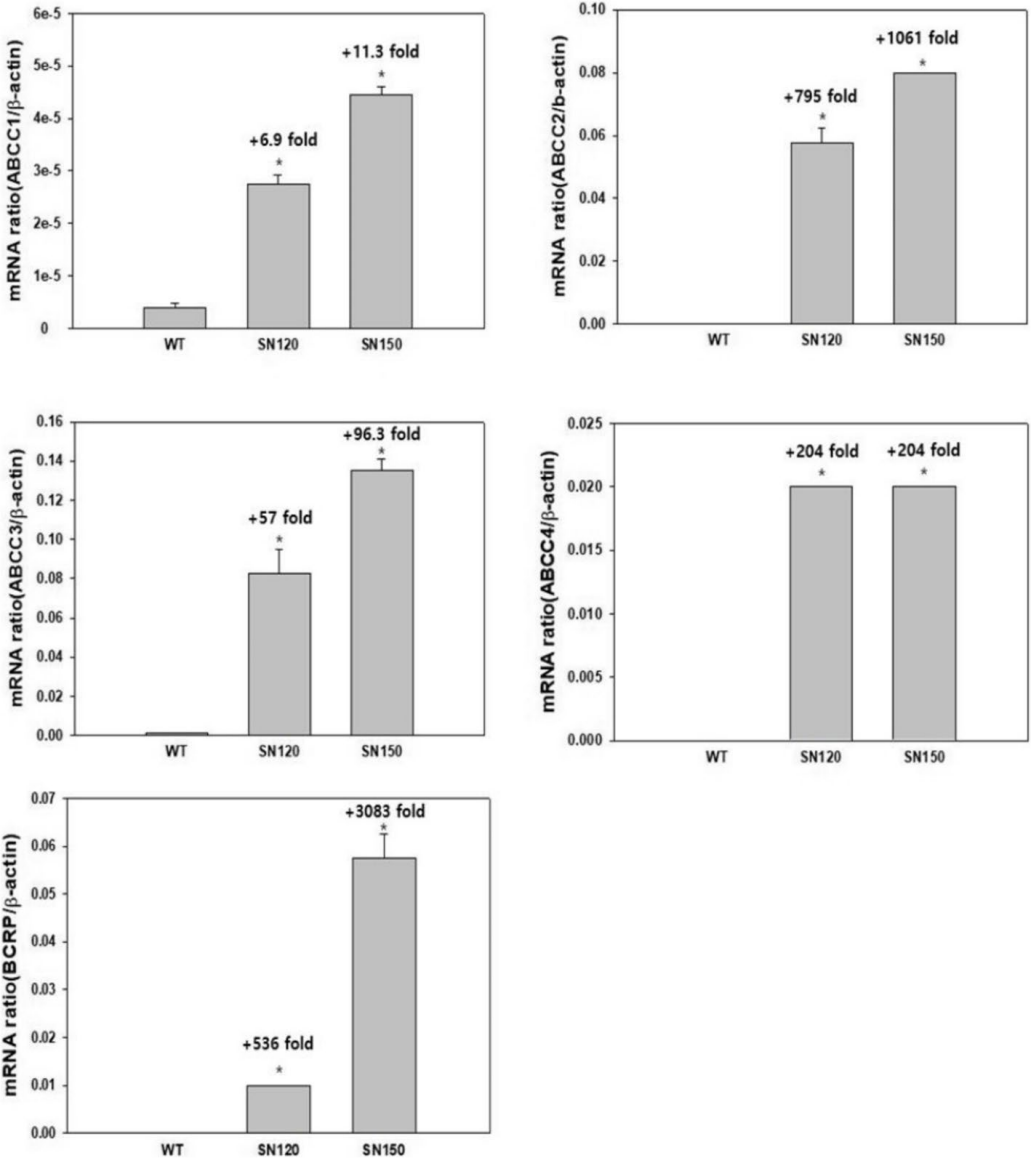
Reverse transcription quantitative PCR assay for MRP1, MRP2, MRP3, MRP4 and BCRP mRNA. RT-qPCR confirmed that T47D/SN120 and T47D/SN150 cells overexpressed *MRP1* (7-fold and 11-fold, respectively), *MRP2* (795-fold and 1061-fold, respectively), *MRP3* (57-fold and 96-fold, respectively), *MRP4* (204-fold both), and *BCRP* (536-fold and 3083-fold, respectively), compared to T47D/WT cells. Numbers above column refer relative fold increase as compared with WT cells. *, *P* < 0.05 vs WT (*n* = 3)

RT-qPCR confirmed that T47D/SN120 and T47D/SN150 cells overexpressed *MRP1* (7-fold and 11-fold, respectively), *MRP2* (795-fold and 1061-fold, respectively), *MRP3* (57-fold and 96-fold, respectively), *MRP4* (204-fold both), and *BCRP* (536-fold and 3083-fold, respectively), compared to T47D/WT cells (Fig. 1).

**Fig. 2:**
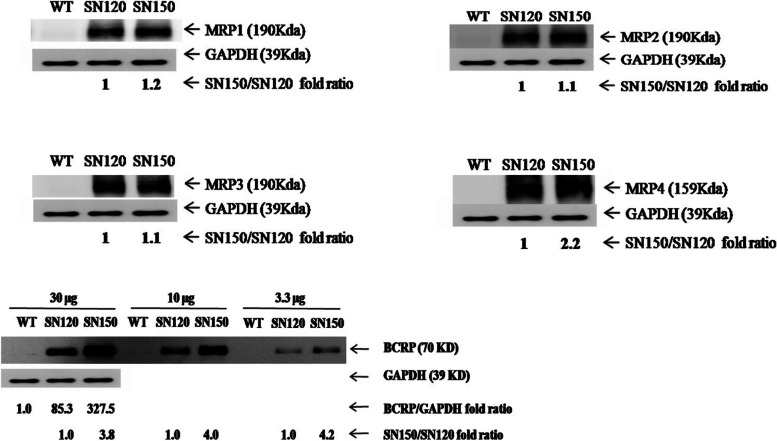
Western blot analysis for MRP1, MRP2, MRP3, MRP4 and BCRP protein. *MRP1, MRP2, MRP3,* and *MRP4* expression levels in T47D/SN150 cells were compared with those in T47D/SN120 cells. T47D/SN150 cells overexpressed *MRP4* (2.2-fold) and *BCRP* (4-fold) protein. Compared to T47D/WT cells, which expressed a trace amount of *BCRP*, T47D/SN120 and T47D/SN150 cells displayed 85.3-fold and 327.5-fold higher *BCRP* protein expression, respectively. (*n* = 3)

Expression levels of *MRP1, MRP2, MRP3, MRP4*, and *BCRP* were determined by western blot analysis. *MRP1, MRP2, MRP3,* and *MRP4* expression levels in T47D/SN150 cells were compared with those in T47D/SN120 cells. Expression levels of *MRP4* and *BCRP* but not *MRP1, MRP2* and *MRP3* were significantly different between both resistant sublines (Fig. 2, Supplementary Fig. 3).

Compared to T47D/WT cells, which expressed a trace amount of *BCRP*, T47D/SN120 and T47D/SN150 cells displayed 85.3-fold and 327.5-fold higher *BCRP* protein expression, respectively. T47D/SN150 cells displayed 4-fold higher expression of *BCRP*, as compared to T47D/SN120 cells (Fig. 2).

### Sensitivity of SN38-resistant T47D sublines to SN38 in the presence of various chemosensitizers

**Fig. 3:**
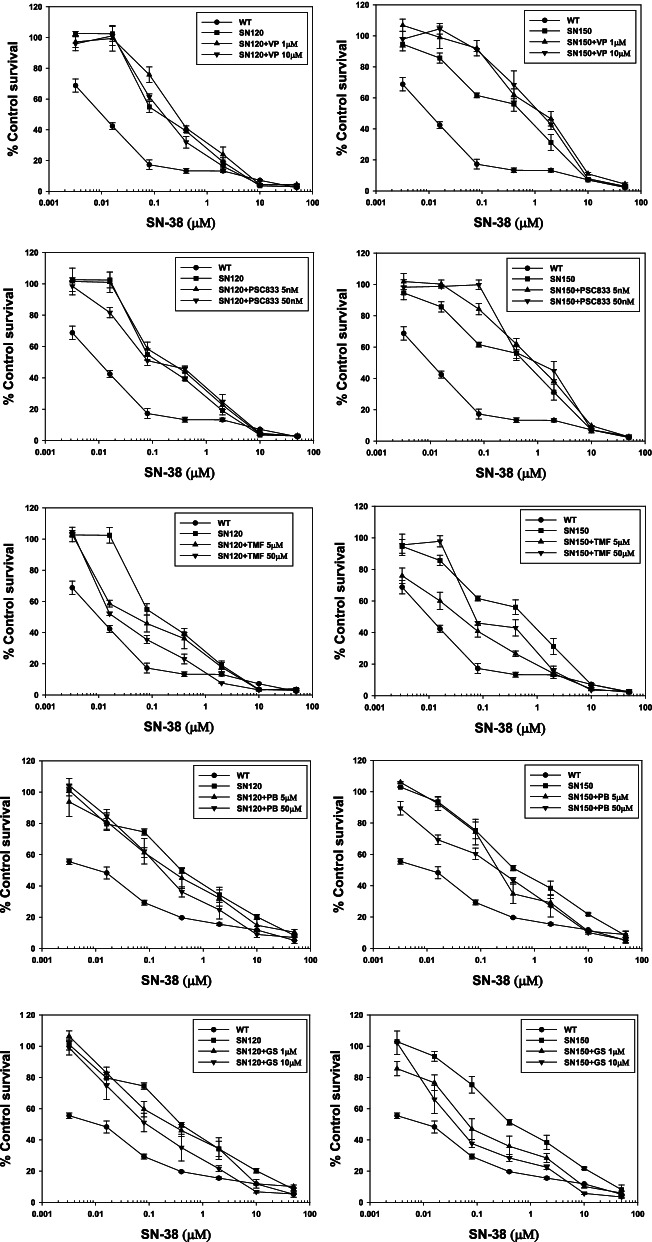
Sensitivity of SN38-resistant T47D sublines to SN38 in the presence of various chemosensitizers. The effects of several chemosensitizers, including *Pgp* [verapamil, PSC833, and 7,3′,4-trimethoxyflavone (TMF)], *MRP* (probenecid), and *BCRP* (genistein) inhibitors, were assessed on SN38-resistant T47D sublines. Among the *Pgp* inhibitors, only TMF showed chemosensitizing effects on SN38 in a concentration-dependent manner. Probenecid and genistein also sensitized SN38-resistant T47D sublines to SN38 in a concentration-dependent manner. (*n* = 3). Vp, verapamil; TMF, 7,3′,4-trimethoxyflavone; PB, probenecid; GS, genistein

The effects of several chemosensitizers, including Pgp [verapamil, PSC833, and 7,3′,4-trimethoxyflavone (TMF)], MRP (probenecid), and BCRP (genistein) inhibitors, were assessed on SN38-resistant T47D sublines [49]. Among the Pgp inhibitors, only TMF showed chemosensitizing effects on SN38 in a concentration-dependent manner. Probenecid and genistein also sensitized SN38-resistant T47D sublines to SN38 in a concentration-dependent manner (Fig. 3). The chemosensitizing effects of probenecid were similar between T47D/SN120 and T47D/SN150 cells whereas those of TMF and genistein in the T47D/SN150 cells was more sensitive as compared to the T47D/SN120 cells. As shown in Table 3, the chemosensitizing index was calculated by dividing the IC_50_ value in the absence of the chemosensitizer with that in its presence. Chemosensitizing indices of probenecid (5 and 50 μM) and genistein (1 and 10 μM) were 1.3–2.0 and 1.2–10.8, respectively, in both the cell lines, whereas those for verapamil (1 and 10 μM) and PSC833 (5 and 50 nM) were less than 1.0, with the exception of PSC833 in the T47D/SN120 cell line, which displayed a chemosensitizing index of 1.3.

**Table 3:** Effect of chemosensitizers on SN38-resistant T47D sublines

	Chemosenensitizing index of chemosensitizers									
	verapamil (μM)		PSC833 (nM)		TMF (μM)		Probenecid (μM)		Genistein (μM)	
	1	10	5	50	5	50	5	50	1	10
SN120	0.56	0.88	0.67	1.30	3.01	7.73	1.31	1.72	1.28	3.90
SN150	0.48	0.50	0.65	0.60	15.95	10.56	2.00	1.99	7.53	10.78

$$\mathrm{Chemosensitizing}\;\mathrm{index}=\frac{{\mathrm{IC}}_{50}\;\mathrm{of}\;\mathrm{SN}38\;\mathrm{in}\;\mathrm{the}\;\mathrm{absence}\;\mathrm{of}\;\mathrm{chemosensitizer}}{{\mathrm{IC}}_{50}\;\mathrm{of}\;\mathrm{SN}38\;\mathrm{in}\;\mathrm{the}\;\mathrm{presence}\;\mathrm{of}\;\mathrm{chemosensitizer}}$$

### Involvement of epigenetic gene silencing of MRP1, MRP2, MRP3, MRP4, and BCRP in T47D/WT cells

**Fig. 4:**
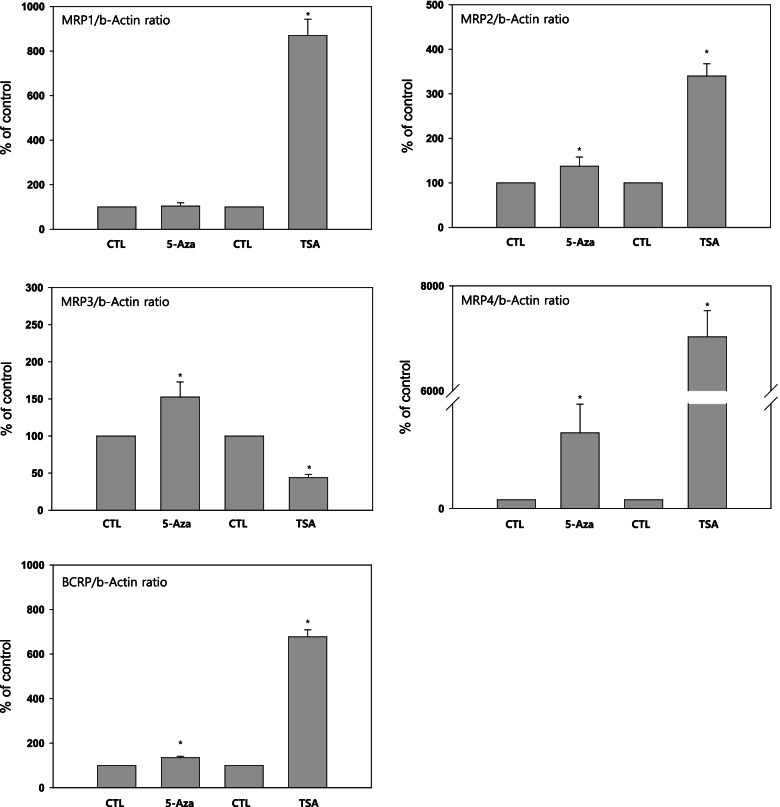
Effects of 5-aza-2′-deoxcytidine (AdC) and trichostatin A (TSA) on expression of MRP1, MRP2, MRP3, MRP4 and BCRP in T47D/WT cells. RT-qPCR indicated that 5-aza-2′-deoxycytidine induced the mRNA expression of *MRP2, MRP3, MRP4,* and *BCRP*, while TSA induced the mRNA expression of *MRP1, MRP2, MRP4*, and *BCRP* in T47D/WT cells. *, *P* < 0.05 vs CTL (*n* = 4)

Next, we investigated whether *MRP1, MRP2, MRP3, MRP4*, and *BCRP* could be epigenetically induced in T47D/WT cells following treatment with 2.5 μM 5-aza-2′-deoxycytidine for 96 h or 100 ng/mL trichostatin A (TSA) for 48 h. RT-qPCR indicated that 5-aza-2′-deoxycytidine induced the mRNA expression of *MRP2, MRP3, MRP4*, and *BCRP*, while TSA induced the mRNA expression of *MRP1, MRP2, MRP4*, and *BCRP* in T47D/WT cells (Fig. 4).

### Decreased fluorescent dye accumulation in T47D/SN120 and T47D/SN150 cells

**Fig. 5:**
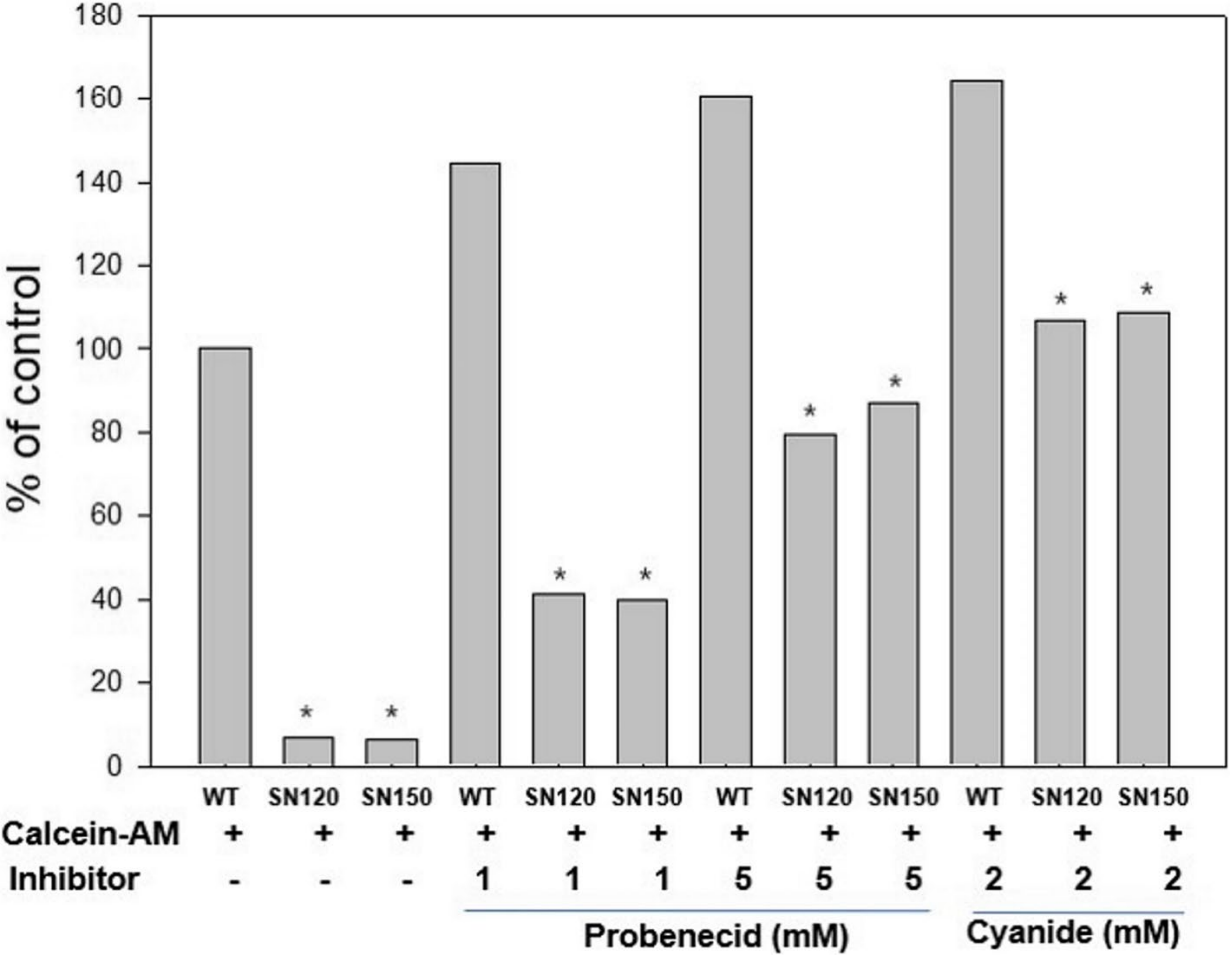
Intracelluar calcein-AM accumulation of the T47D/WT, T47D/SN120 and T47D/SN150 cells. Accumulation of calcein AM decreased in T47D/SN120 and T47D/SN150 cells, compared to that in T47D/WT cells. However, calcein AM accumulation was increased after treatment with MRP inhibitor (probenecid) and ATP deplete cyanide. *, *P* < 0.05 vs WT (*n* = 3)

**Fig. 6:**
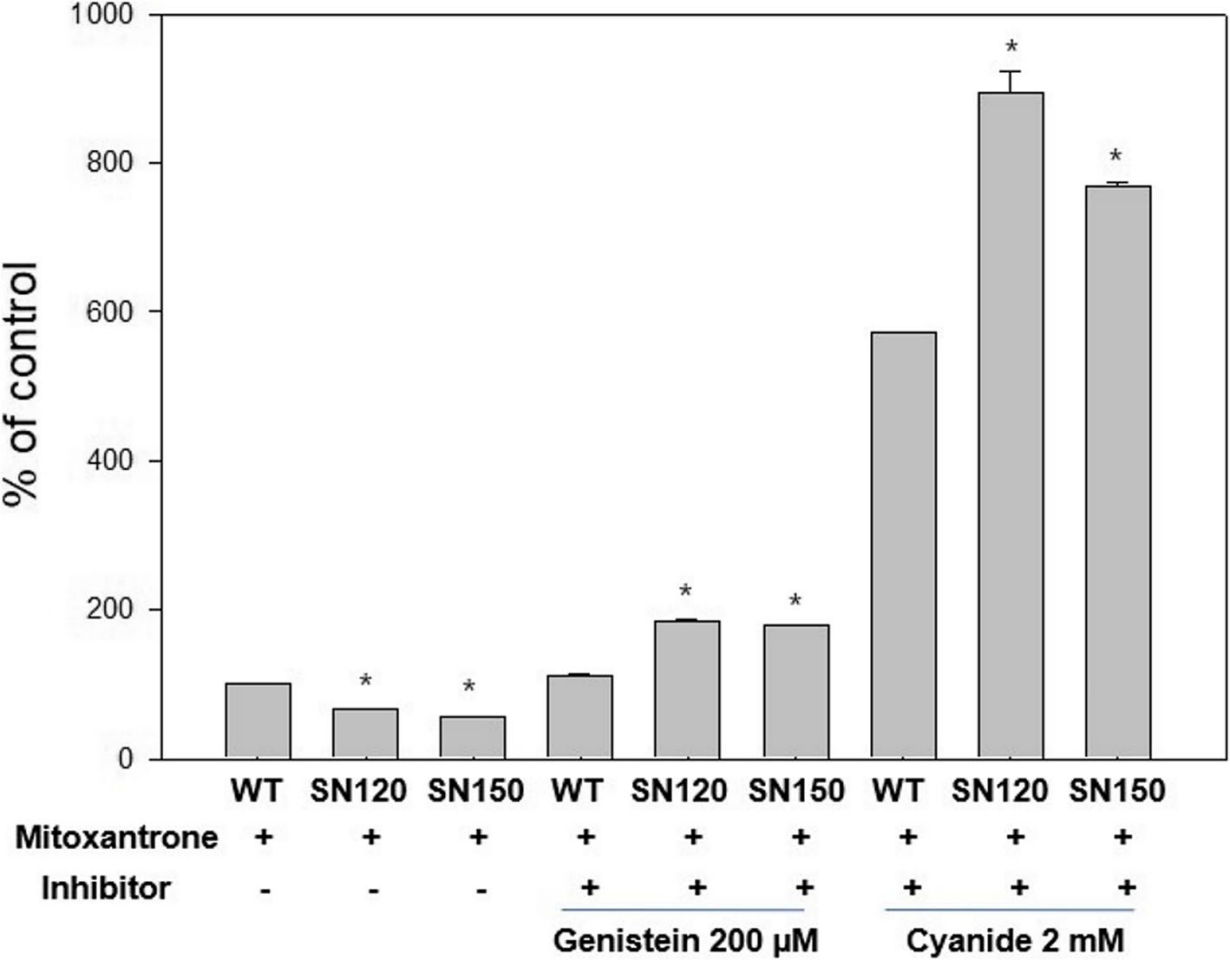
Intracelluar mitoxantrone accumulation of the T47D/WT, T47D/SN120 and T47D/SN150 cells. Mitoxantrone accumulation decreased in T47D/SN120 and T47D/SN150 cells, compared to that in T47D/WT cells. However, mitoxantrone accumulation was increased after treatment with BCRP inhibitor (genistein) and ATP deplete cyanide. *, *P* < 0.05 vs WT (*n* = 3)

Fluorescent dye accumulation was assayed to estimate the functional activity of transporters in T47D/SN120 and T47D/SN150 cells. Calcein AM and mitoxantrone were used as fluorescent substrates for MRP and BCRP, respectively, and detected using flow cytometry. Accumulation of both the substrates decreased in T47D/SN120 and T47D/SN150 cells, compared to that in T47D/WT cells. However, upon treatment with the inhibitors probenecid and genistein, there was an increase in the accumulation of both the substrates (Figs. 5-6).

Meanwhile, intracellular levels of rhodamine 123, a fluorescent substrate of *Pgp*, were not affected by treatment with a *Pgp* inhibitor PCS833 in T47D/SN120 and T47D/SN150 cells (Supplementary Fig. 4).

## Discussion

Although there is continuous development of new strategies for breast cancer prognosis and treatment, chemotherapy resistance is frequently encountered and remains a major obstacle in the management of breast cancer. Eventually, MDR develops in most systemic recurrent or initially metastatic breast cancer patients. Overcoming MDR could significantly improve the efficacy of chemotherapy.

The aim of this study was to establish new resistant breast cancer cell lines that could aid the development of novel chemosensitizers to eventually overcome MDR and to investigate MDR mechanisms.

We established two new resistant breast cancer cell sublines, T47D/SN120 and T47D/SN150. Both sublines showed a wide variety of resistance to anticancer drugs tested in this study from huge resistance (paclitaxel and vinblastine) to low or no resistance (5-FU and methotrexate).

Generally, the levels of resistance are classified as high (RF >20x), moderate (RF 5-15x), and low or no resistance (RF <5x) [50]. Considering that common substrates for both *MRPs* and *BCRP* are SN38, irinotecan and methotrexate, and substrates for *MRP* only are paclitaxel, vinblastine and cisplatin and those for *BCRP* only are doxorubicin, topotecan, gefitinib, mitoxantrone [51–53], the resistance profiles of both resistant sublines to anticancer drugs seems to be closely related to the expression levels of transporters although there are some exceptions which remain to be determined.

Compared to their parental cell line, T47D/SN120 and T47D/SN150 cells displayed faster proliferation and a shorter doubling time. These features were not consistent with other resistant cell lines such as MCF-7/TAX [54], BEL-7402/5-FU [55], MCF-7/Doc, and MCF-7/Adr [36]. This may be a result of the short incubation period. Most cells follow an S-shaped pattern of growth; however, we calculated the doubling time at a time point prior to the S-shaped growth curve.

The mRNA expression profiles of the transporter genes in T47D/SN120 and T47D/SN150 cells were compared to those in the T47D/WT cell line. The T47D/WT did not express *Pgp* and *MRP1* mRNAs, which had already been reported in another study [56]. We found that T47D/SN120 and T47D/SN150 cells overexpress MRP1, MRP2, MRP3, MRP4, and BCRP. However, we did not perform RNA sequencing although a number of splicing variants of MPR mRNA had been reported [57]. Moreover, T47D/SN150 cells obtained following exposure to a higher concentration of SN38 expressed a higher level of transporter genes associated with MDR. This suggests that MDR is related to the drug concentration used as well as overexpression of MDR-related genes.

MTT assays were performed to assess the sensitivity of SN38-resistant T47D sublines to SN38 in the presence of inhibitors (*Pgp* inhibitors: verapamil, PSC833, and TMF; *MRP* inhibitor: probenecid; *BCRP* inhibitor: genistein) [49]. TMF, probenecid, and genistein showed a concentration-dependent chemosensitizing effect. The *MRP* and *BCRP* expression in T47D/SN150 cells was higher than that in T47D/SN120 cells and the chemosensitizing effects of TMF and genistein were more effective in T47D/SN150 than that in T47D/SN120 cell lines. Interestingly, administration of the well-known *Pgp* inhibitors, verapamil and PSC833 did not sensitize the cells to SN38; however, the sensitivity was recovered upon administration of TMF. In addition, the sensitivity recovery pattern observed in SN38-resistant breast cancer cells treated with TMF was similar to that observed in SN38-resistant breast cancer cells treated with genistein. This suggests that TMF not only functions as a *Pgp* inhibitor, but also affects other MDR mechanisms. The inhibitory effects of TMF on non-*Pgp* transporters remain to be investigated.

Next, experiments were designed to identify the relationship between the expression of *MRP1, MRP2, MRP3, MRP4*, and *BCRP* and epigenetic gene silencing. Generally, alteration of gene function in cancer is attributed to either genetic alterations (such as mutations or deletions) or epigenetic alterations, which alter the gene expression status [58, 59]. Epigenetic alterations include promoter methylation and chromatin remodeling, such as histone modification without an alteration in the DNA sequence [60, 61]. Changes in gene expression caused by epigenetic alterations in cancer can be divided into three categories, transcriptional regression by methylation of the promoter CpG islands (lesions in the genome rich in sequences consisting of a cytosine preceding a guanine), increased gene expression by hypomethylation, and decreased gene expression associated with histone deacetylase (HDAC) [62–64]. Aberrant DNA methylation in normally unmethylated gene promoter CpG islands results in a decrease in gene expression. Hypermethylation of promoter CpG islands in tumor suppressor genes is a hallmark of all human cancers [65–67]. Moreover, global hypomethylation of the DNA contributes to carcinogenesis by inducing genomic instability [68–70]. DNA hypomethylation is associated with activation of proto-oncogenes [71]. Lastly, activated HDAC induces promoter DNA methylation and represses gene expression [58, 64, 72]. Consequently, epigenetic gene silencing occurs by DNA hypermethylation and histone deacetylation. Therefore, DNA methyltransferase inhibitors and HDAC inhibitors, which inhibit DNA methylation and histone deacetylation, induce the expression of genes that were abnormally suppressed. Thus, they have been used as therapeutic agents to treat cancer caused by epigenetic gene silencing. In this study, expression of MRP2, MRP3, MRP4, and BCRP were found to be significantly increased in T47D/WT cancer cells treated with the DNA methyltransferase inhibitor, 5-aza-2′-deoxycytidine. Similarly, treatment of T47D/WT cancer cells with the HDAC inhibitor, trichostatin A significantly increased the expression of *MRP1*, *MRP2, MRP4*, and *BCRP*. In other words, contrary to what occurs in cancer induced by epigenetic gene silencing, overexpression of *MRP1, MRP2, MRP3, MRP4*, and *BCRP* might be caused by DNA demethylation and histone acetylation. These results suggest that the expression of *MRP1, MRP2, MRP3, MRP4*, and *BCRP* could be induced by the suppression of epigenetic gene silencing. These findings are consistent with reports indicating that epigenetic gene silencing is involved in irinotecan sensitivity in colorectal cancer cells [73–75]. Intracellular irinotecan is inactivated by UGT1A1 or pumped out by transporters such as *ABCB1* (*Pgp*), *ABCC1* (*MRP1*), and *ABCC2* (*MRP2*) [76, 77]. Since silencing of UGT1A1 and transporters occurs via DNA methylation, DNA methyltransferase inhibitors can restore gene expression and thereby, enhance SN38 inactivation [74]. ABC transporter gene silencing occurs through histone deacetylation [73, 75] . In this study, surprisingly, the expression of MRP3 decreased after treatment with TSA; however, the underlying mechanism remains to be elucidated and is difficult to explain because studies on the epigenetic profile of the ABC loci are still incomplete. Also, other possible mechanisms of overexpression of ABC transporters includes gene amplification and post-transcriptional regulations by miRNAs [78–80].

*BCRP, MRP1, MRP2, MRP3*, and *MRP4* decreased the intracellular fluorescent dye accumulation. When the *BCRP* and *MRP* inhibitors genistein and probenecid were added as chemosensitizers, the intracellular fluorescent dye concentrations were found to be increased. However, addition of the *Pgp* inhibitor PSC833 did not increase the intracellular fluorescent dye accumulation, suggesting that the resistance of T47D/SN120 and T47D/SN150 cells to anti-cancer drugs is not associated with *Pgp*.

## Conclusions

Two SN38-resistant breast cancer cell sublines, T47D/SN120 and T47D/SN150, were established by gradual exposure to SN38. The resistant cells overexpressed MRP1, MRP2, MRP3, MRP4, and BCRP, which might be due to the suppression of epigenetic gene silencing via DNA hypermethylation and histone deacetylation. These resistant cells presented classical MDR phenotype, characterized by cross-resistance to various other anti-cancer drugs. This MDR phenotype decreased intracellular drug accumulation, which was reversed by known MRP and BCRP chemosensitizers, probenecid and genistein, respectively. Thus, the both SN-38-resistant sublines overexpressing both MRP and BCRP are valuable for screening chemosensitizers inhibiting both transporters.

## Supplementary Information

**Additional file 1: Supplemental Figure 1**.

**Additional file 2: Supplemental Figure 2**.

**Additional file 3: Supplemental Figure 3**.

**Additional file 4: Supplemental Figure 4**.

## Data Availability

The datasets used during the current study are available from the corresponding author on reasonable request.

## Abbreviations

BCRP: Breast cancer resistance protein
SN38: 7-ethyl-10-hydroxycamptothecin
MTT: 3-(4,5-methylthiazol-2-yl)-2,5-diphenyl-tetrazolium bromide
MDR: Multidrug resistance
ABC: ATP (adenosine triphosphate)-binding cassette
MRP1: Multidrug resistance protein 1
MRP2: Multidrug resistance protein 2
MRP3: Multidrug resistance protein 3
MRP4: Multidrug resistance protein 4
LRP: Lung resistance protein
RF: Resistance factor
RT-qPCR: Reverse transcription-quantitative polymerase chain reaction
PBS: Phosphate-buffered saline
TMF: 7,3′,4-trimethoxyflavone
Pgp: P-glycoprotein
TSA: Trichostatin A
HDAC: Histone deacetylase
