# Supplementary figures and images for: Characterization of SN38-resistant T47D breast cancer cell sublines overexpressing BCRP, MRP1, MRP2, MRP3, and MRP4
Source: BMC Cancer. 2022 Apr 23;22:446. doi: 10.1186/s12885-022-09446-y (PMC9035251; doi:10.1186/s12885-022-09446-y)

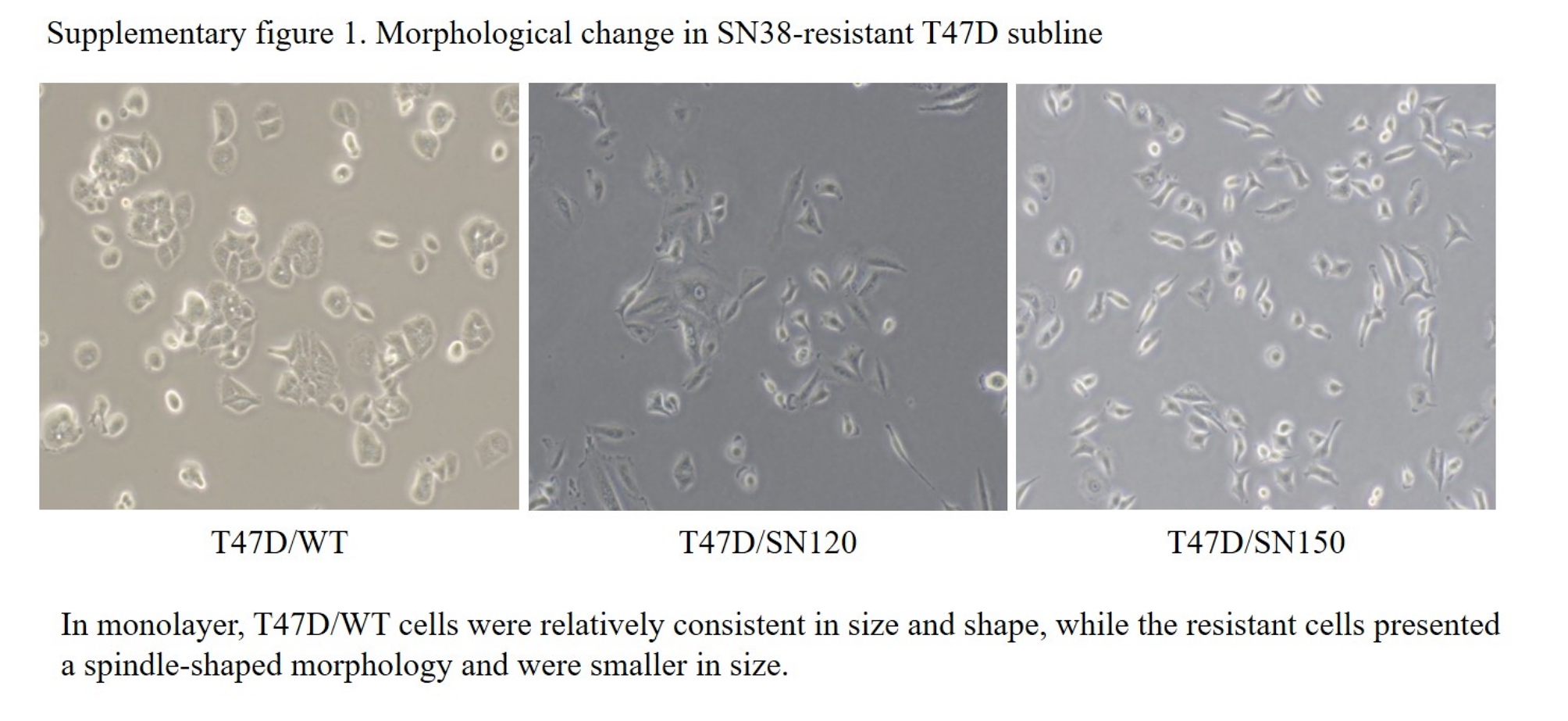

Supplement: Supplementary file 1 — Additional file 1: Supplemental Figure 1. [file 12885_2022_9446_MOESM1_ESM.jpg]

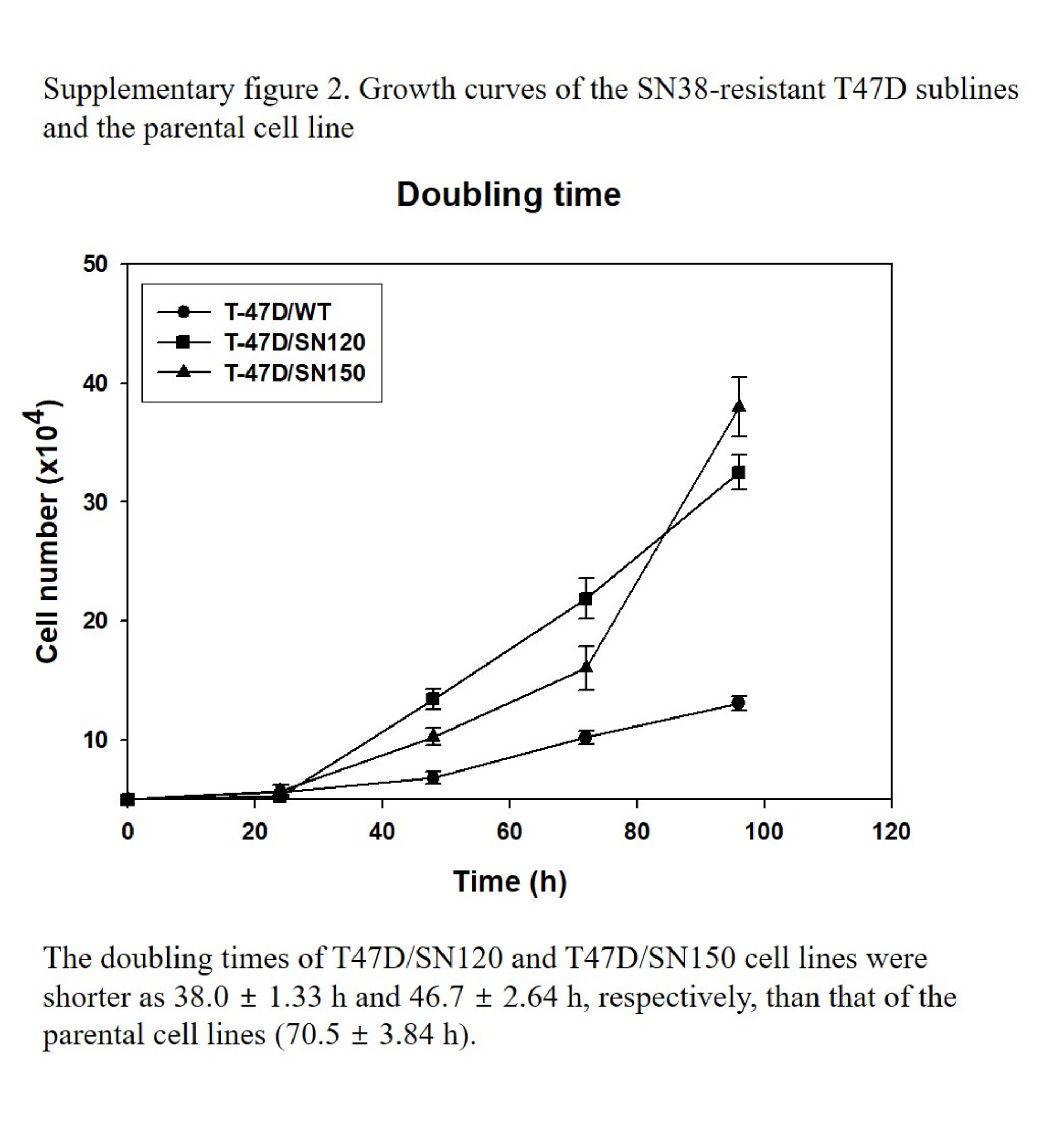

Supplement: Supplementary file 2 — Additional file 2: Supplemental Figure 2. [file 12885_2022_9446_MOESM2_ESM.jpg]

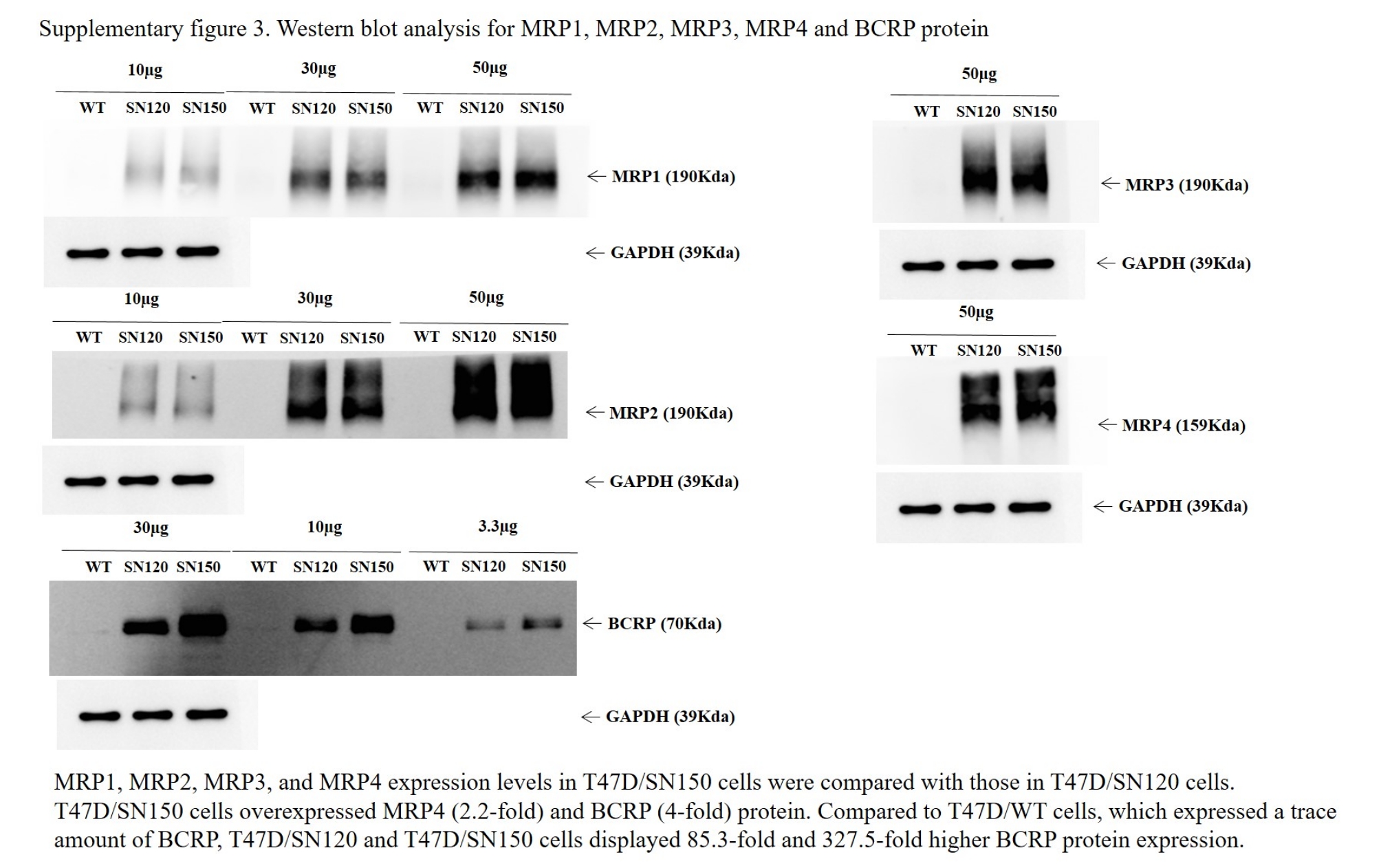

Supplement: Supplementary file 3 — Additional file 3: Supplemental Figure 3. [file 12885_2022_9446_MOESM3_ESM.jpg]

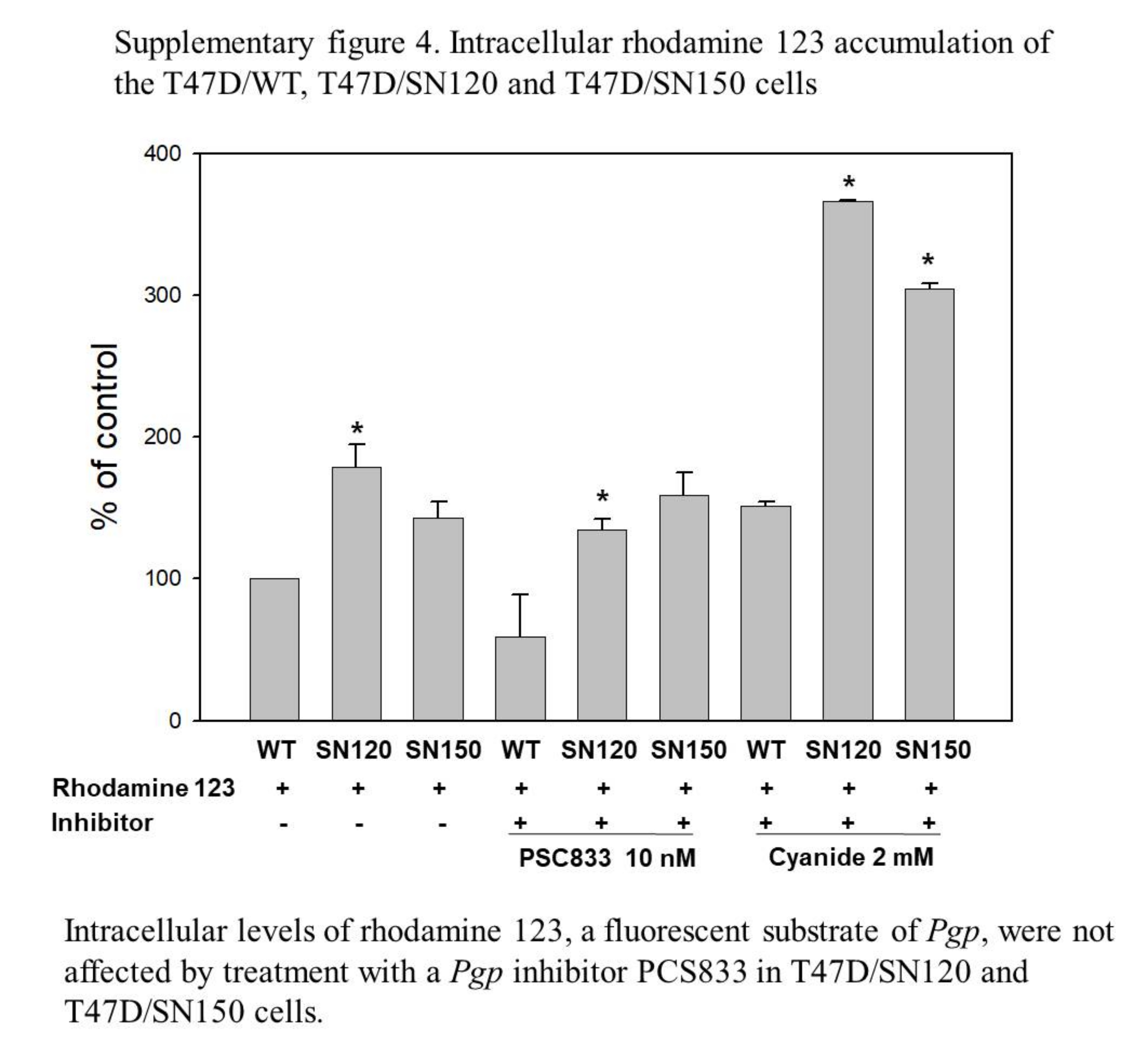

Supplement: Supplementary file 4 — Additional file 4: Supplemental Figure 4. [file 12885_2022_9446_MOESM4_ESM.jpg]
